# The relationship between TMCO1 and CALR in the pathological characteristics of prostate cancer and its effect on the metastasis of prostate cancer cells

**DOI:** 10.1515/biol-2022-0972

**Published:** 2024-10-29

**Authors:** Jingting Dong, Shaosan Kang, Fenghong Cao, Xi Chen, Xiaofei Wang, Lei Wang, Qing Wang, Yupu Zhai

**Affiliations:** Department of Urology, Affiliated Hospital of North China University of Science and Technology, 73 Jianshe Nan Lu, Lubei District, Tangshan, Hebei, 063000, P.R. China; Pathology Department, Affiliated Hospital of North China University of Science and Technology, Tangshan, Hebei, 063000, China; Department of Urology, Luannan County Hospital, Tangshan, Hebei, 063000, China; Department of Urology, Fengnan Hospital of Traditional Chinese Medicine, Tangshan, Hebei, 063000, China

**Keywords:** prostate cancer, TMCO1, CALR, metastasis

## Abstract

Calcium homeostasis is correlated closely with the occurrence and development of various cancers. The role of calcium homeostasis in prostate cancer has remained unclear. The present study aimed to investigate the relationship between transmembrane and crimp-crimp domain 1 (TMCO1) and calreticulin (CALR) in the pathological characteristics of prostate cancer and the mechanism of action on prostate cancer metastasis. Effects of CALR recombinant protein and TMCO1 knockdown on prostate cancer cells were investigated using following methods: cell cloning, Transwell, wound scratch assay, JC-1 assay, Fluo-4 Assay, endoplasmic reticulum (ER) fluorescent probe, mitochondrial fluorescence probe, Western blot and Immunofluorescence. TMCO1 and CALR are overexpressed in prostate cancer and knockdown of TMCO1 significantly inhibited the invasion, migration and cell proliferation. Furthermore, knocking down TMCO1 modulated the intensity of ER probes and mitochondrial fluorescence probes, and affected the levels of intracellular calcium ion and mitochondrial membrane potential. In addition, CALR recombinant protein upregulated the expression of epithelial-mesenchymal transition marker, Vimentin, Conversely, knockout of TMCO1 significantly reduced the expression of CALR and Vimentin. Knockout of TMCO1 can reverse the effect of CALR recombinant protein, elucidating the pivotal roles of TMCO1 and CALR in the regulation of prostate cancer metastasis through modulation of calcium homeostasis.

## 1 Introduction

Globally, prostate cancer (PCa) is the most prevalent malignancy among men, with a pronounced incidence in developed countries [1], and ranks within the top five cancers for both incidence and mortality worldwide, contributing significantly to cancer-related mortality among elderly men [2]. The standard therapeutic approaches for PCa include drug treatment and surgical intervention. However, a substantial proportion of patients face the challenges of recurrence and metastasis [3]. The majority of PCa-related mortalities are attributed to the metastatic spread of tumor cells, which can disseminate to various distant organs such as the lungs, liver, bones, and lymph nodes [4].

Transmembrane and coiled-coil domains 1 (TMCO1), also known as endoplasmic reticulum (ER) Ca^2+^ Load-Activated Ca^2+^ Channel, is an ER transmembrane protein [5]. Studies have shown that deletion or downregulation of the TMCO1 gene can perturb intracellular calcium balance, triggering ER stress and aberrant Ca^2+^ signaling, resulting in tumorigenesis [6]. Calreticulin (CALR) serves as the primary calcium-binding protein in the ER and exhibits multifaceted roles in cellular function [7]. CALR impedes the binding of the androgen receptor to hormone-responsive DNA elements, thereby suppressing the transcriptional activity of retinoic acid receptors. This inhibition also affects the neural differentiation processes induced by retinoic acid [8]. Elevated levels of CALR have been observed in various malignancies, including gastric, lung, and pancreatic cancers [9]. Despite these findings, it is still unclear how the interaction between TMCO1 and CALR influences PCa metastasis.

Therefore, we propose a hypothesis that TMCO1 and CALR influence ER calcium regulation and mediate prostate cancer cell metastasis. Our study delineates the interplay between TMCO1 and CALR, elucidates their association with the pathological characteristics and prognostic indicators of prostate cancer, and lays a theoretical groundwork for the development of targeted therapeutic interventions.

## 2 Materials and methods

### 2.1 TCGA data analysis

The Cancer Genome Atlas (TCGA; http://gepia.cancer-pku.cn/index.html) is a comprehensive database that encompasses a vast array of prostate cancer (PCa) samples coupled with rich clinical data [10]. Leveraging TCGA, we conducted an analysis of the gene expression profiles of TMCO1 and CALR in PCa tissues and normal tissues.

### 2.2 Clinical sample collection

After obtaining written informed consent, specimens were collected from PCa patients in the Affiliated Hospital of North China University of Science and Technology. The study was approved by the ethics committees of each participating center. From January 2021 to December 2023, we collected 59 prostate biopsy specimens, electroprostatectomy specimens and total prostatectomy specimens of PCa patients in the Affiliated Hospital of North China University of Science and Technology. And we collected the relevant clinicopathological data.


**Informed consent:** Informed consent has been obtained from all individuals included in this study.
**Ethical approval:** The research related to human use has been complied with all the relevant national regulations, institutional policies and in accordance the tenets of the Helsinki Declaration, and has been approved by the Ethical Committee of Affiliated Hospital of North China University of Technology (202103001).

### 2.3 Cell culture and transfection

Human PCa cell lines (DU145 and PC-3) were come from National Collection of Authenticated Cell Cultures. DU145 cells were cultured in Dulbecco’s Modified Eagle’s Medium appended with 10% FBS and 1% penicillin–streptomycin. PC-3 cells maintained in F-12K medium supplemented with 10% FBS, 1% penicillin-streptomycin. All cells were maintained at 37°C in a humidified atmosphere of 95% air and 5% CO_2_.

Cells were seeded into six-well plates, and upon reaching a confluence of 70–80%, 150pmol siRNA and Lipofectamine 8000 were added and transfected for 24 h at 37°C according to the manufacturer’s protocol. Small interfering RNAs were purchased from Sangon Biotech Co. Post-transfection efficiency was evaluated using Western blot analysis. The sequences were as follows: TMCO1 siRNA1(Sense: 5′-CAGGCAUAACCUGGGUCCUGGUUUA-3′; antisense: 5′-UAAACCAGGACCCAGGUUAUGCCUG-3′); TMCO1 siRNA2(Sense: 5′-UAGAGAGACAAGAAGAGAAACUGAA-3′; antisense: 5′-UUCAGUUUCUCUUCUUGUCUCUCUA-3′)

TMCO1 siRNA3(Sense: GGGUCCUGGUUUACAGGACAGACAA; antisense: 5′-UUGUCUGUCCUGUAAACCAGGACCC-3′); negative control (Sense: 5′-GGGUCGGUUAUACGGCAGAACUCAA-3′; antisense: 5′-UUGAGUUCUGCCGUAUAACCGACCC-3′).

### 2.4 Immunofluorescence

Tissues were routinely dehydrated and embedded in paraffin, and serial sections were dewaxed to water; EDTA antigen repair solution was heated. The primary antibody TMCO1 (1:200, ab238768, Abcam, USA;), CALR (1:100, ab92516, Abcam, USA) was incubated overnight at 4°C. After rewarming for 60 min at room temperature, the sections were incubated with a secondary antibody (1:100, ZF-0316, ASGB-BIO, China) at 37°C for 60 min. Add 1–2 drops of fluorescent blocking tablets (including DAPI) to seal the tablets, then observe under a fluorescence microscope.

### 2.5 Immunohistochemical

Tissues were routinely dehydrated and embedded in paraffin; serial sections were dewaxed to water, and EDTA antigen repair solution was heated. The primary antibody TMCO1 (1:200, ab238768, Abcam, USA;) and CALR (1:100, ab92516, Abcam, USA) was incubated overnight at 4°C. Incubated for 60 min at room temperature for rewarming. Incubation with secondary antibody (PV-9001, ASGB-BIO, China) was at 37°C for 1 h. The newly prepared DAB chromogenic hematoxylin staining solution was dropped and counterstained. Finally, dehydration and sealing were done. Immunohistochemical staining was blindly scored by two pathologists.

### 2.6 CCK8

Cell Counting Kit-8 was used to evaluate cell viability. Simply, DU145 and PC-3 human PCa cells were seeded in 96-well plates (1,000 cells/well) for 24 h. Subsequently, 10 μL of the CCK-8 reagent was added to each well, and the plates were incubated for 3 h, strictly following the manufacturer’s guidelines. The OD value at 450 nm was measured.

### 2.7 Transwell

Following a 24-h serum starvation period, DU145 and PC-3 cells were prepared into a cell suspension, and 200 µL of the cell suspension was added to the Transwell upper chamber (50,000 cells/well). The culture was conventionally grown at 37°C and 5% CO_2_ for 24 h. Subsequently, the cells were fixed with 4% paraformaldehyde solution for 10 min and stained with 0.1% crystal violet for 20 min. Subsequently, the cells were washed twice with PBS and gently wiped with a cotton swab to remove residual cells. Five visual fields were randomly selected from the center and surrounding areas; these fields were then observed, photographed, and counted with an inverted microscope.

### 2.8 Wound scratch assay

Cells of DU145 and PC-3 in a logarithmic growth phase, at a density of 40,000 cells/well, were plated and cultured until confluence exceeded 90%. On the backside of the 6-well plate, horizontal lines were evenly marked at 1 cm intervals across the well using a marker pen. Pipette tips were used to scratch two parallel lines in the central area where the cells grew. The cells were washed with PBS for 3 times to remove the scratched cells. Samples were taken at 0 h time point, photographed at 100× magnification, and the samples were placed into an incubator at 37°C with 5% CO_2_ for further cultivation. Samples were taken at 24 h time point, and photographs were taken at 100×.

### 2.9 Cell cloning

DU145 and PC-3 cells in the logarithmic growth phase were harvested at a density of 300 cells per well and resuspended in a culture medium. The cell suspension was then diluted in a series of gradient multiples. The cells were maintained in an incubator for 2 weeks. Subsequently, cells were fixed with 4% paraformaldehyde for 15 min. After fixation, the solution was aspirated, and the cells were stained with a 0.1% crystal violet solution for 20 min. Following staining, the cells were washed twice with PBS and allowed to air dry.

### 2.10 ER fluorescent probe

Intracellular ER levels were measured utilizing specific ER-Tracker Red fluorescent probes (C1041, Beyotime, https://www.beyotime.com/product/C1041.htm). The cell culture medium was removed, and the ER-Tracker Red working solution, pre-warmed to 37°C, was introduced. The cells were then incubated with the staining solution at 37°C for 25 min. Post incubation, the ER-Tracker Red solution was removed, and the cells were gently washed twice with fresh cell culture fluid. The cells were finally observed under a fluorescence microscope.

### 2.11 Mitochondrial fluorescence probe

A minute quantity of the 200 μM Mito-Tracker Red CMXRos (C1049B, Beyotime, https://www.beyotime.com/product/C1049B-50%CE%BCg.htm) stock solution was diluted in the cell culture medium at a ratio of 1:10,000, yielding a final concentration of 200 nM. Then, the cells were incubated with this working solution at 37°C for 25 min. Following the incubation period, the Mito-Tracker Red CMXRos working solution was aspirated, and the cells were then replenished with fresh, pre-warmed cell culture medium maintained at 37°C. The cells were subsequently examined under a fluorescence microscope.

### 2.12 Mitochondrial membrane potential

In accordance with the manufacturer’s instructions for reagent (C2003S, Beyotime, https://www.beyotime.com/product/C2003S.htm), first, the JC-1 staining working solution was prepared by pipetting 5 µL of JC-1 (200×) from the kit and mixing it thoroughly with 1 mL of JC-1 staining buffer. The cells were seeded into a six-well plate, adding 1 mL of the JC-1 staining solution to each well. Then, the cells were incubated with JC-1 staining solution at 37°C for 20 min. After incubation, the staining solution was removed and the cells were washed twice with 1 mL of JC-1 staining buffer per well. The Carbonyl cyanide 3-chlorophenyl hydrazone (CCCP), supplied at a concentration of 10 mM in the kit, was introduced to the cell culture medium at a dilution ratio of 1:1,000, resulting in a final concentration of 10 μM. Then, CCCP was incubated with cells for 20 min. The application of CCCP served as the positive control in the experiment. Finally, the stained cells were observed under a fluorescence microscope.

### 2.13 Fluo-4 calcium assay kit

The medium was removed, and the cells were washed three times with HBSS solution. Fluo4-AM (S1060, Beyotime, https://www.beyotime.com/product/S1060.htm) working solution was added, and the amount of solution was subject to the covering cell. The cells were incubated at 37°C for 30 min in a cell incubator. Cells were washed three times with HBSS solution. Then HBSS solution was added to cover the cells. The cells were incubated for approximately 30 min at 37°C in an incubator to ensure to facilitate the complete conversion of Fluo-4 AM into Fluo-4. The cells were visualized under a fluorescence microscope, and representative images were captured.

### 2.14 Western blot

Cells were washed with PBS, and protein lysate was prepared for lysis. After centrifugation, the supernatant was taken as whole protein extract and stored at −80°C. After quantification of the BCA protein, the protein was denatured, and SDS–PAGE gel electrophoresis was prepared. The separated proteins were transferred to the PVDF membrane. Subsequently, primary antibodies TMCO1 (1:1,000, 27757-1-AP, Proteintech, China), CALR (1:1,000, ab92516, Abcam, USA), Vimentin (1:2,000, ab92547, Abcam, USSA), GAPDH (1:3,000, ab8245, Abcam, USA), and β-actin (1:1,000, ab8226, Abcam, USA) were added and incubated at 4°C overnight. Then, they were incubated with secondary antibodies (1:5,000, ZB-2301, ASGB-BIO, China) or (1:5,000; ZB-2305; ASGB-BIO, China). The images were developed in the gel imaging instrument, and the images were saved.

### 2.15 Co-immunoprecipitation (Co-IP)

Following the protocol of the Immunoprecipitation Kit with Protein A + G Magnetic Beads (P2179, Beyotime), the cells were lysed by the addition of 100 μL of lysis buffer. The lysate was then centrifuged at 10,000 × *g* at 4°C for 5 min, and the supernatant was collected; 50 μL was reserved as the input control. Both the 3 μg antibody and an equivalent amount of normal IgG, serving as negative controls, were diluted according to the manufacturer’s instructions for the antibodies. These were then incubated with 100 μL of Protein A + G magnetic beads at room temperature for 1 h to allow the beads to bind to the antibody. A volume of 500 μL of cell lysate was added to the Protein A + G magnetic beads that had been bound with either antibodies or normal IgG. This mixture was incubated on a shaker at room temperature overnight at 4°C. After incubation, the mixtures were centrifuged at 1,000*g* at 4°C for 5 min, and the supernatant was removed; 100 μL of SDS-PAGE Sample Loading Buffer (1×) was added, and the mixture was heated at 95°C for 5 min. The supernatant was then collected for Western detection using the method described in Section 2.14.

### 2.16 Statistical analysis of data

SPSS 22.0 was used for statistical analysis, and all measurement data were expressed in the form of mean ± standard deviation. Differences between two groups were compared using Student’s *t*-test analysis of variance. One-way analysis of variance was used to determine the differences between three or more groups. *P* < 0.05 was considered to indicate a statistically significant difference. All experiments were performed independently at three times.

## 3 Results

### 3.1 TMCO1 and CALR may be the key genes affecting PCa metastasis

**Figure 1:**
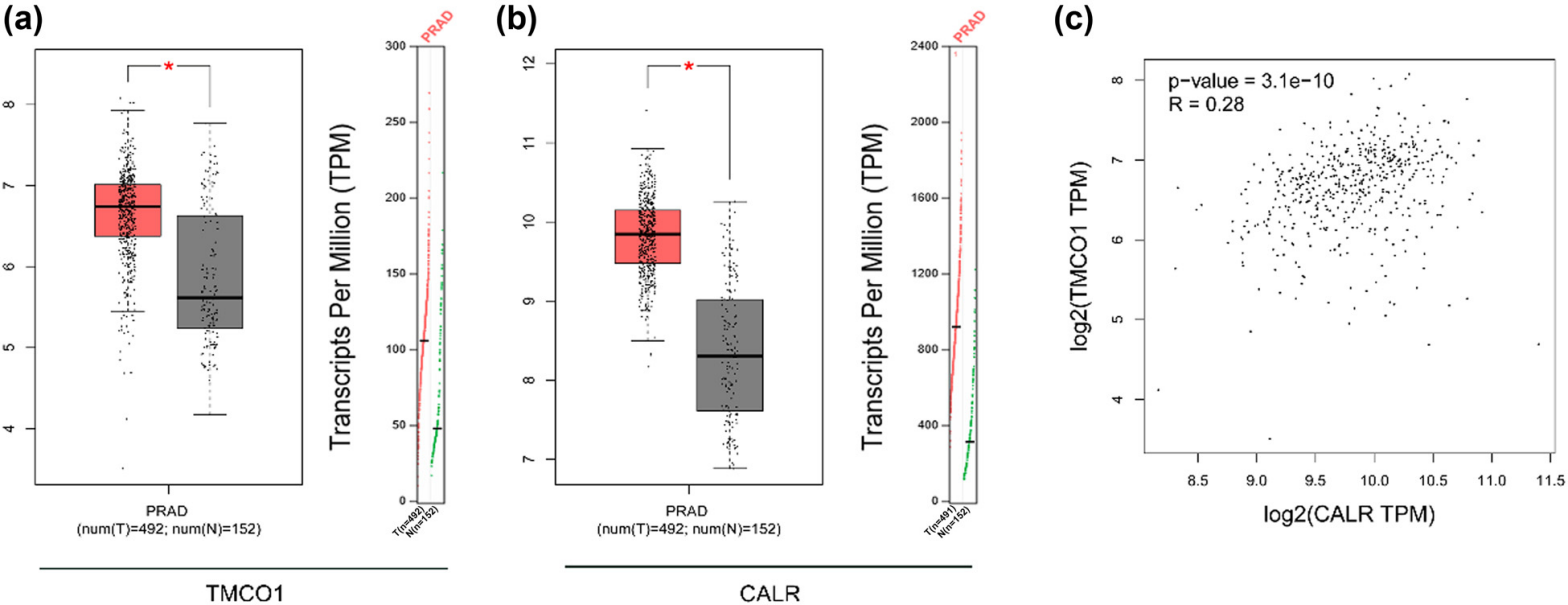
TCGA data analysis TMCO1 and CALR. (a) TCGA data analysis of TMCO1 expression specificity. In the box figure, the red is the expression of TMCO1 in cancer tissue, and the black is the expression of TMCO1 in normal prostate tissue. (b) TCGA data analysis of CALR expression specificity. In the box figure, red is the expression of CALR in cancer tissue, and black is the expression of CALR in normal prostate tissue. (c) Correlation between TMCO1 and CALR expression was analyzed by TCGA data. PRAD is PCa. *P < 0.05.

To determine the expression pattern of TMCO1 and CALR in PCa, we compared its expression in PCa and normal tissues using the TCGA data. As shown in Figure 1a and b, the expression level of TMCO1 and CALR were significantly higher in prostate cancer samples than in normal samples. Furthermore, a correlation analysis was performed to examine the relationship between TMCO1 and CALR, revealing a significant association between these two proteins (Figure 1c).

### 3.2 TMCO1 and CALR are highly expressed in PCa samples

**Figure 2:**
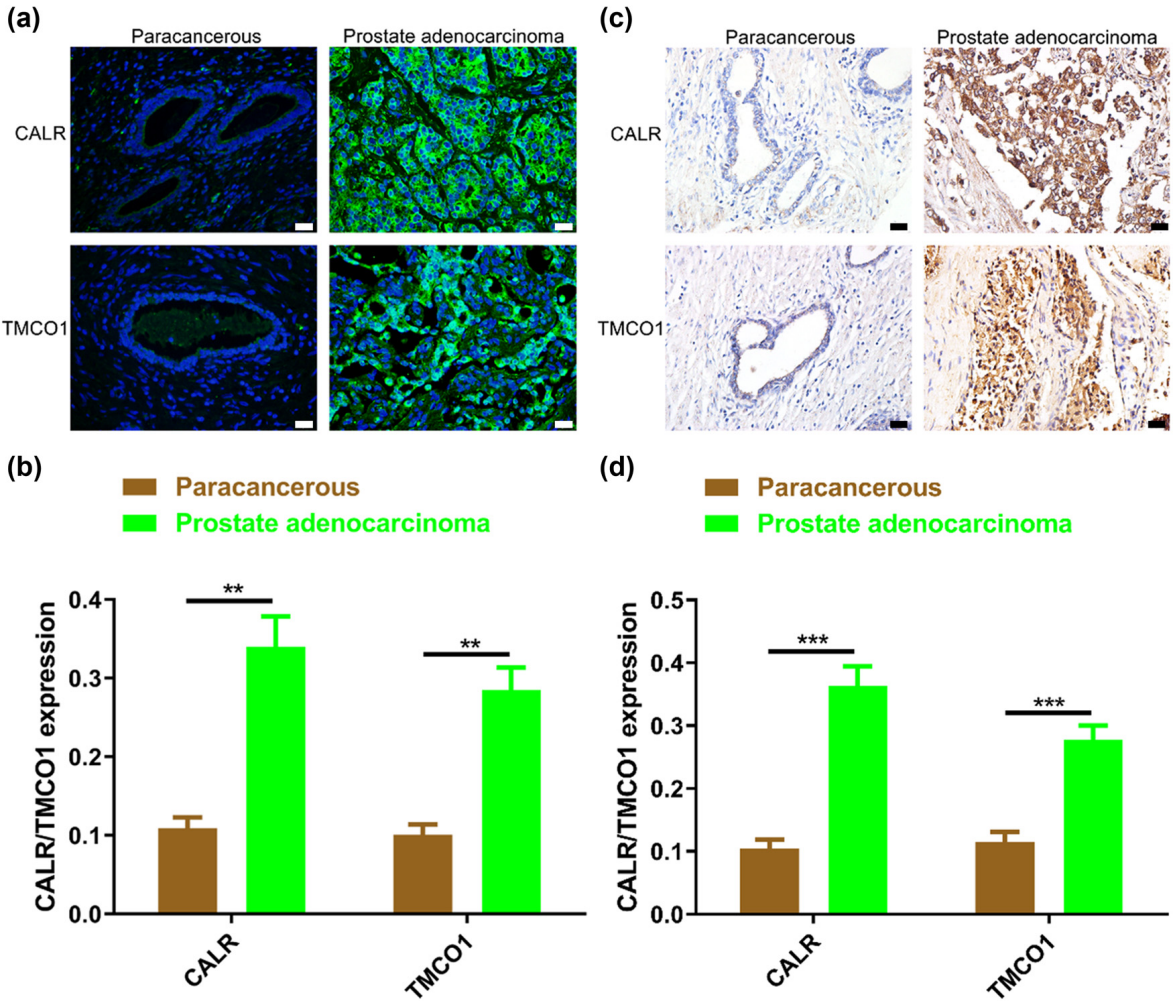
Expression of TMCO1 and CALR in PCa. (a) and (b) Immunofluorescence showed that CALR and TMCO1 genes were differentially expressed in adjacent and prostatic adenocarcinoma tissues, respectively. (c) and (d) Immunohistochemistry showed that the protein expressions of CALR and TMCO1 genes were different in adjacent and prostatic adenocarcinoma tissues, respectively. Scale bars, 20 μm. **P < 0.01, ***P < 0.001.

We collected a total of 59 prostate biopsy specimens, electro prostatectomy specimens, total prostatectomy specimens, and along with pertinent clinicopathological data. Immunofluorescence and immunohistochemistry showed that TMCO1 and CALR were highly expressed in PCa tissues (Figure 2a–d), a finding that corroborated the analysis of the TCGA database. The expressions of TMCO1 and CALR were correlated with the Gleason score defined by WHO. Based on the analysis of the relationship between clinicopathologic features, TMCO1 was closely related to lymphatic metastasis, invasion depth, clinical stage and survival (Table 1).

**Table 1:** Expression and clinicopathological features of TMCO1 and CALR in Pca

Characteristics	Total (*n* = 59)	TMCO1		CALR	
		Positive (*n* = 37)	Negative (*n* = 22)	Positive (*n* = 45)	Negative (*n* = 14)
**Age (year)**					
≤60	24	16	8	18	6
＞60	35	21	14	27	8
*χ* ^*2*^		0.271	0.036		
*P*-value		0.603	0.849		
**Gleason score**					
≤7	26	12	14	15	11
＞7	33	25	8	30	3
*χ* ^*2*^		5.450	8.866		
*P*-value		0.020	0.003		
**T stage**					
T1–2	27	13	14	16	11
T3–4	32	24	8	29	3
*χ* ^*2*^		4.515	7.960		
*P*-value		0.034	0.005		
**Metastasis to lymph nodes**					
Yes	34	26	8	31	3
No	25	11	14	14	11
*χ* ^*2*^		6.496	9.850		
*P*-value		0.011	0.002		
**Invasion to seminal vesicle**					
Yes	39	28	11	34	5
No	20	9	11	11	9
*χ* ^*2*^		4.059	5.891		
*P*-value		0.044	0.015		
**AJCC clinicopathological stage**					
I–IIc	16	7	9	8	8
IIIa–IIIc	28	23	5	24	4
IVa–IVb	15	7	8	13	2
*χ* ^*2*^		8.633	7.358		
*P*-value		0.013	0.026		
Survival					
Yes	32	16	16	21	11
No	27	21	6	24	3
*χ* ^*2*^		4.832	4.379		
*P*-value		0.028	0.036		

### 3.3 Optimal interference sequence of recombinant protein CALR and TMCO1

**Figure 3:**
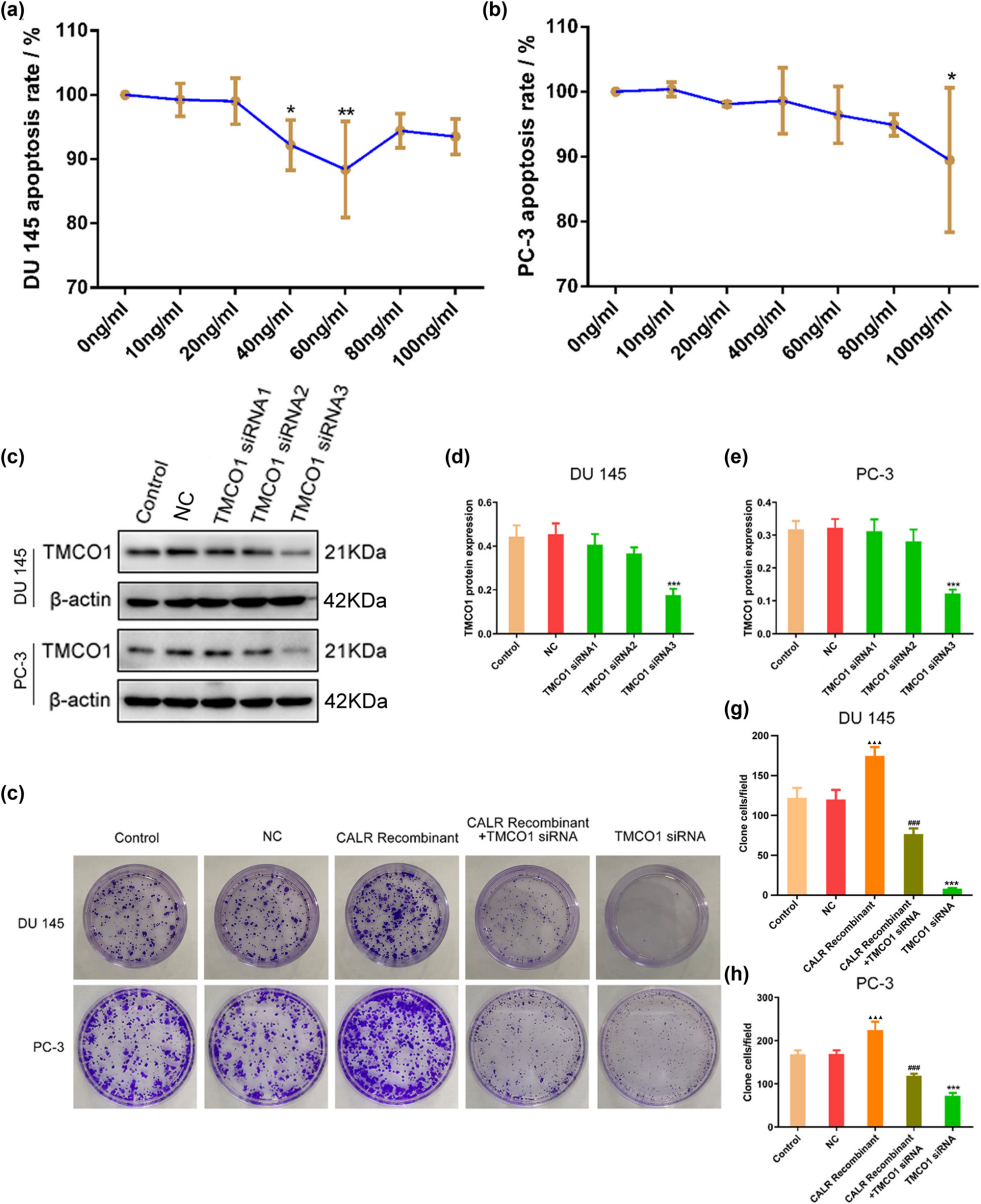
(a) and (b) Detection of the effect of CALR human recombinant protein on the proliferation of DU145 and PC-3 human PCa cells. (c)–(e) Screening the optimal interference sequence of TMCO1 in DU145 and PC-3 human PCa cells. (f)–(h) Effects of TMCO1 siRNA, CALR recombinant protein and TMCO1 siRNA + CALR recombinant protein on colony formation of DU145 and PC-3 cells. ***P < 0.001 vs NC, ^###^ *P* < 0.001 vs CALR Recombinant, ^▲▲▲^ *P* < 0.001 vs Control.

To determine the optimal concentration of CALR recombinant protein, CCK-8 assay was performed to examine cell proliferation of PC-3 and DU145 cells treated with a series of concentrations of CALR recombinant protein. Our data showed that when the concentration of CALR recombinant protein reached 20 ng/mL, the effect on cell apoptosis was minimal. Hence, 20 ng/mL CALR recombinant protein was chosen for the experimental concentration (Figure 3a and b). Three TMCO1 siRNA were synthesized, and their protein expression levels were observed. The Western blot results showed that the transfection effects of TMCO1 siRNA3 were significantly better than TMCO1 siRNA1 and TMCO1 siRNA2. Therefore, TMCO1 siRNA3 was chosen as the interference sequence for use in subsequent experiments (Figure 3c–e).

In a cell cloning experiment, it was found that the proliferation ability of PCa cells was significantly enhanced following the application of CALR recombinant protein. Knockdown of TMCO1 expression significantly inhibited the proliferation ability of prostatic cancer cells, Moreover, the knockdown also mitigated the proliferative impact induced by the CALR recombinant protein (Figure 3f–h).

### 3.4 Knockout of TMCO1 reversed CALR recombinant protein-induced invasion and migration of PCa cells

**Figure 4:**
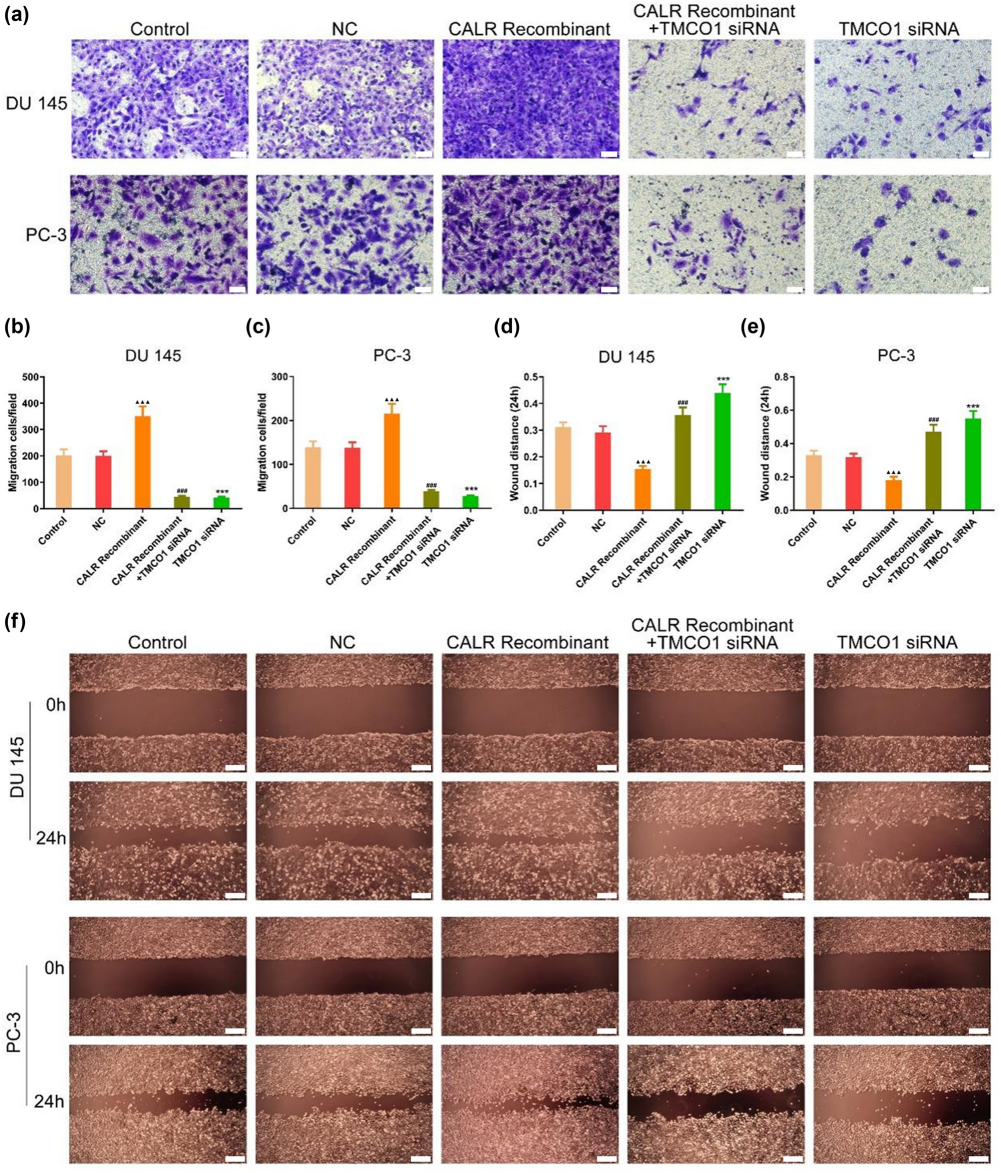
Effects of TMCO1 and CALR on invasion and migration of prostate cancer cells. (a)–(c) Changes in cell invasion ability after TMCO1 siRNA, CALR recombinant protein, TMCO1 siRNA + CALR recombinant protein in DU145 cells. Scale bars, 50 μm. (d)–(f) Changes in cell migration ability after TMCO1 siRNA, CALR recombinant protein, TMCO1 siRNA + CALR recombinant protein in PC-3 cells. Scale bars, 200 μm. ***P < 0.001 vs NC, ^###^ *P* < 0.001 vs CALR Recombinant, ^▲▲▲^ *P* < 0.001 vs Control.

Using the scratch and Transwell assays, we observed a significant enhancement in the invasive and migratory capabilities of DU 145 and PC-3 cells following the application of CALR recombinant protein. Furthermore, the knockout of TMCO1 significantly inhibited the invasion and migration ability of DU 145 and PC3 cells. Compared with the CALR recombinant group, the CALR recombinant + TMCO1siRNA group exhibited a significant decrease in the number of invaded cells and a concurrent increase in the migration area (Figure 4a–f).

### 3.5 TMCO1 and CALR knockdown regulates mitochondrial membrane potential and calcium ion imaging in PCa cells

**Figure 5:**
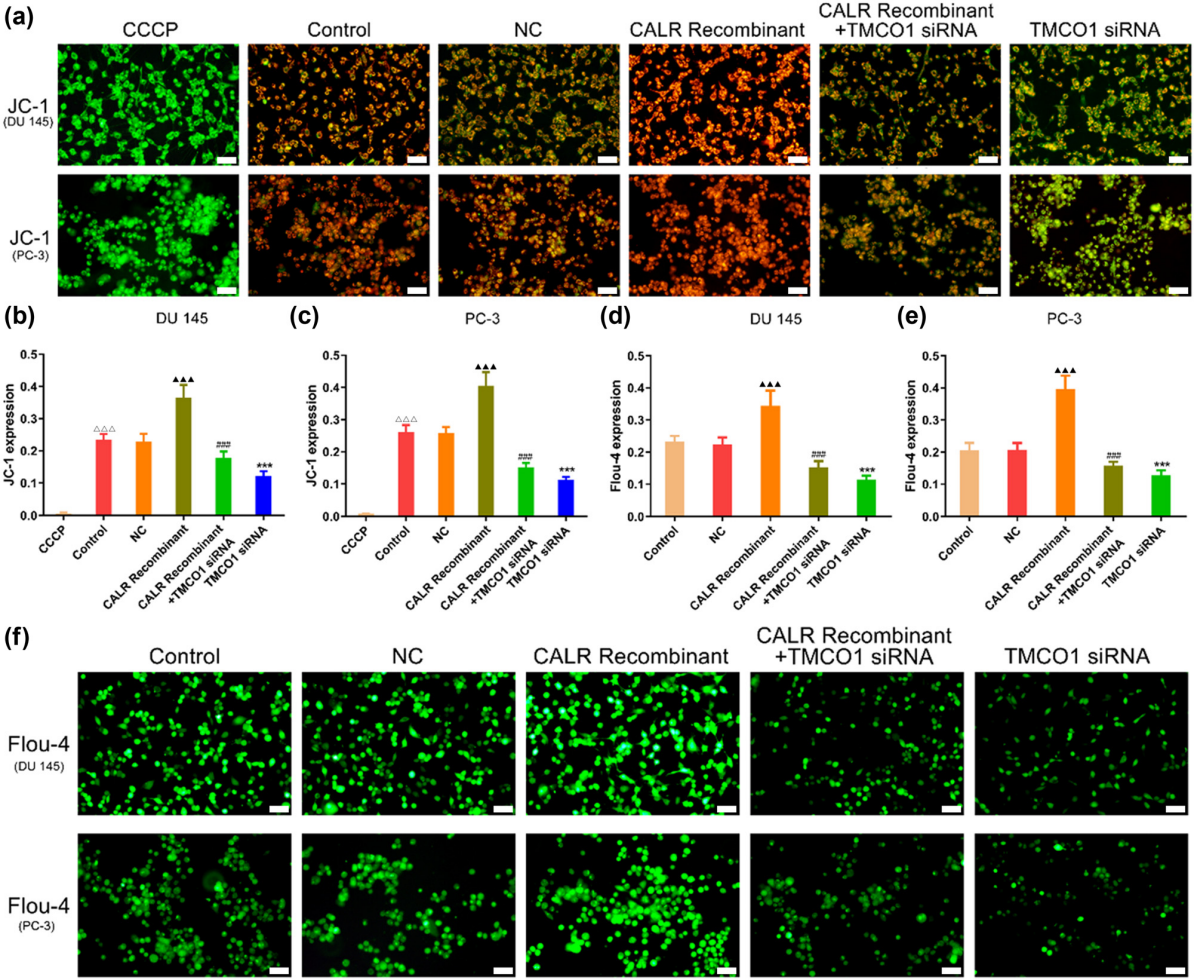
Mitochondrial membrane potential and calcium ion imaging results. (a)–(c) Mitochondrial membrane potential observed in each group on prostate cancer cell (DU145 and PC-3) membrane potential, CCCP is a positive control group. (d)–(f) The effect of each group on calcium level of prostate cancer cells (DU145 and PC-3) was observed by the Fluo4 AM experiment. Scale bars, 50 μm. ***P < 0.001 vs NC, ^###^ *P* < 0.001 vs CALR Recombinant, ^△△△^ *P* < 0.001 vs CCCP, and ^▲▲▲^ *P* < 0.001 vs Control.

Mitochondrial membrane potential assays revealed a significant increase in the intensity of the mitochondrial membrane potential in DU145 and PC-3 cells following the application of CALR recombinant protein, as evidenced by a pronounced enhancement in red fluorescence. Compared with the NC group, the knockdown of TMCO1 significantly reduced the mitochondrial membrane potential in DU145 and PC-3 cells. Furthermore, when compared with the group treated with CALR recombinant protein alone, the mitochondrial membrane potential was significantly lower in the group cotreated with CALR recombinant protein and TMCO1 siRNA. (Figure 5a–c). Calcium imaging assays demonstrated an increase in intracellular calcium ion levels in DU145 and PC-3 cells following the administration of CALR recombinant protein. In contrast, the knockout of TMCO1 significantly diminished the fluorescence intensity in these cells, indicating a reduced presence of intracellular calcium ions. (Figure 5d–f). These findings suggest that TMCO1 and CALR play roles in calcium regulation in prostate cancer.

### 3.6 Interfering with TMCO1 and overexpressing CALR regulate ER and mitochondrial function

**Figure 6:**
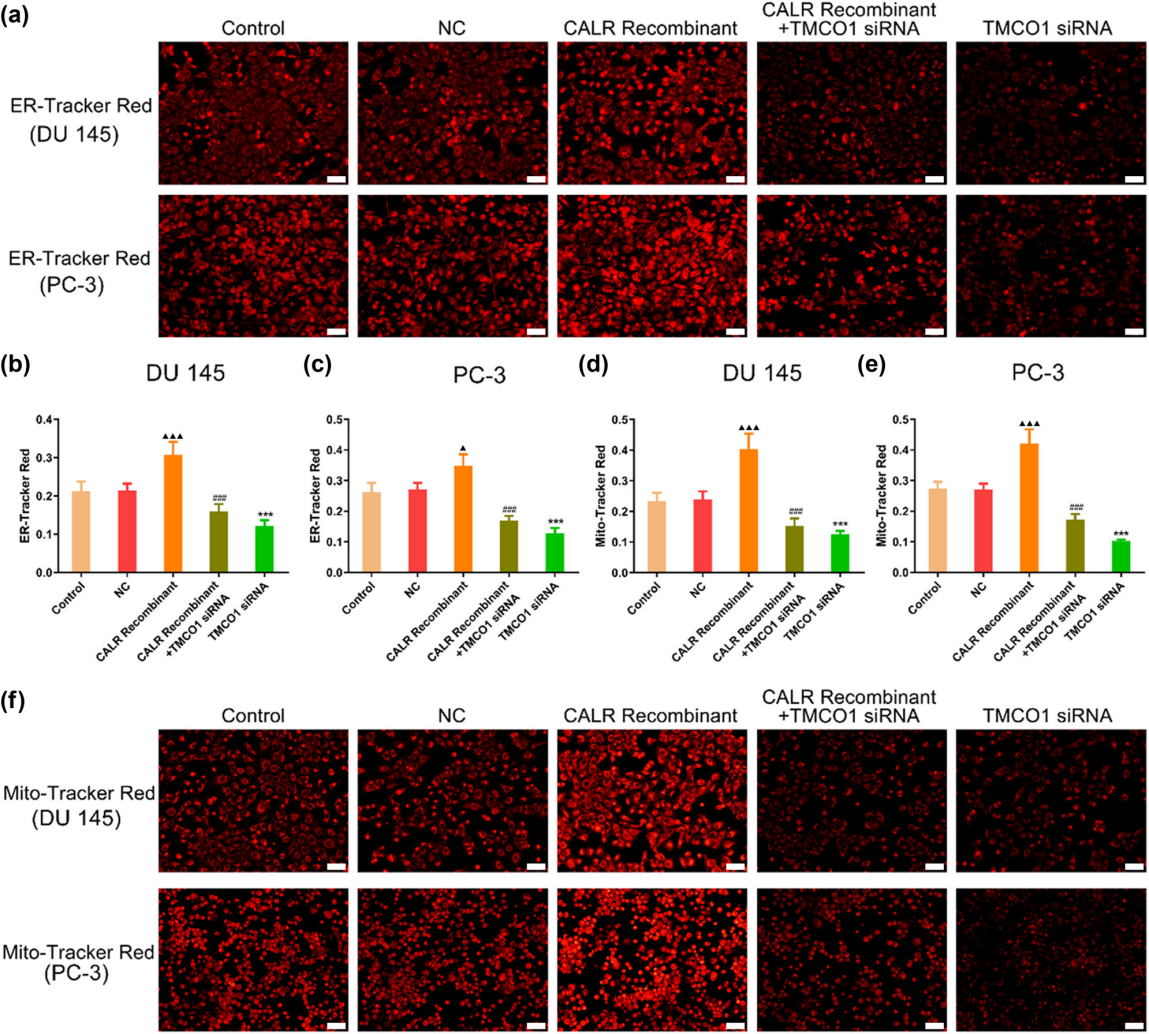
Changes of Prostate Cancer Cells by Endoplasmic Reticulum Probe and Mitochondrial Probe. (a)–(c) ER Tracker Red was used to detect the fluorescence intensity of ER in DU145 and PC-3 cells after TMCO1 siRNA, CALR recombinant protein and TMCO1 siRNA + CALR recombinant protein. (d)–(f) Mitochondrial Tracker Red was used to detect the mitochondrial fluorescence intensity levels of TMCO1 siRNA, CALR recombinant protein and TMCO1 siRNA + CALR recombinant protein in DU145 and PC-3 cells. Scale bars, 50 μm., ***P < 0.001 vs NC, ^###^ *P* < 0.001 vs CALR Recombinant, ^▲^ *P* < 0.05 vs Control, and ^▲▲▲^ *P* < 0.001 vs Control.

Fluorescence probe experiments conducted on five groups of cells revealed a significant increase in the fluorescence intensity of both the ER and mitochondrial probes in DU145 and PC-3 cells following the application of CALR recombinant protein (Figure 6a–c). Conversely, interference with TMCO1 substantially reduced the fluorescence intensity of these probes in DU145 and PC-3 cells (Figure 6d–f), thereby diminishing the induction effect of CALR recombinant protein.

### 3.7 Regulation of Vimentin expression by interfering TMCO1 and CALR recombinant proteins

**Figure 7:**
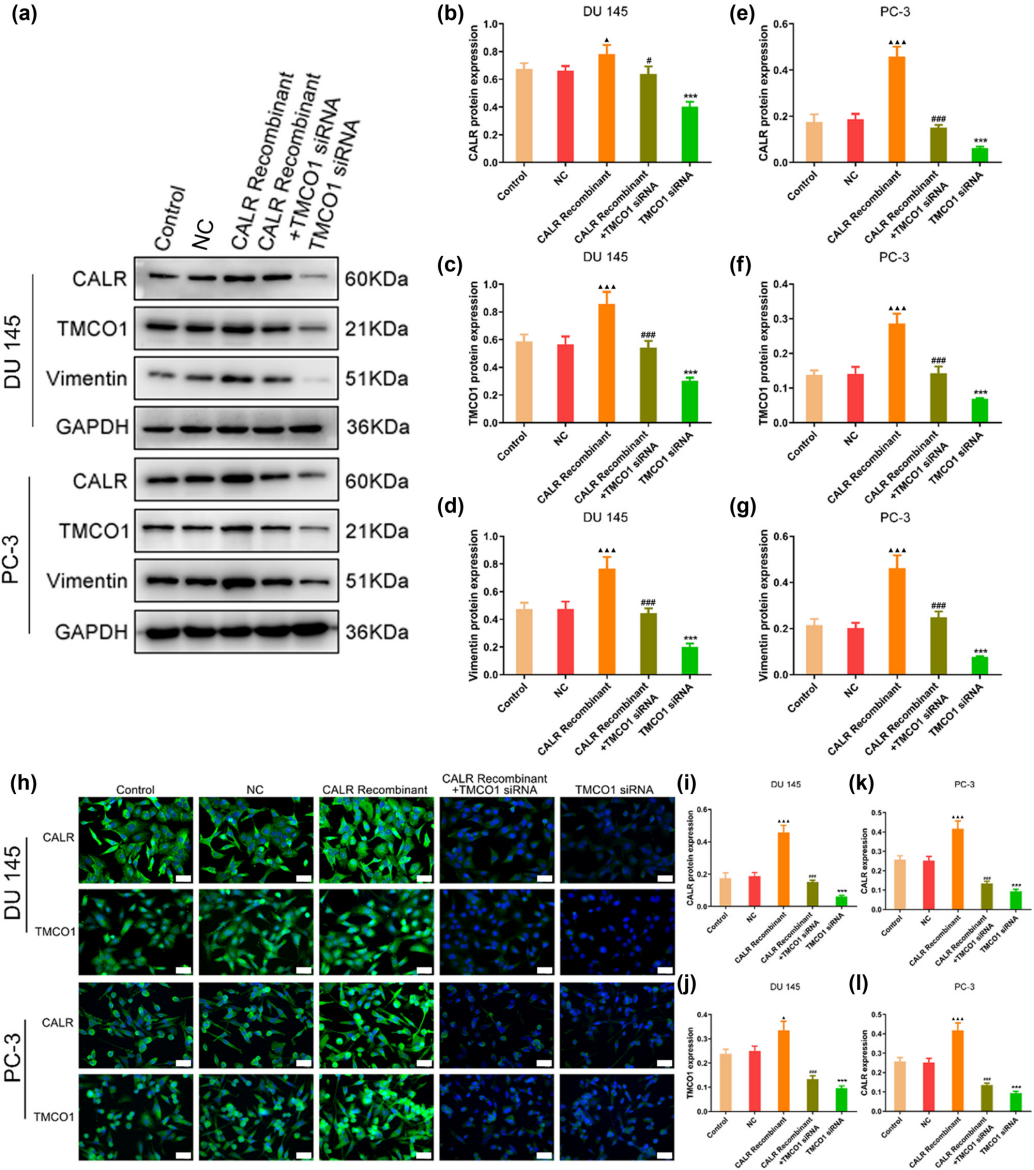
The relationship between TMCO1and CALR in prostate cancer cells. (a)–(g) Western blot was used to detect the effects of TMCO1 and CALR recombinant proteins and their combination on the expression of CALR and Vimentin. (h)–(l) Immunofluorescence assay was used to detect the effects of TMCO1 and CALR recombinant proteins and their combination on the expression of CALR. Scale bars, 20 μm. ***P < 0.001 vs NC, ^#^ *P* < 0.05 vs CALR Recombinant, ^###^ *P* < 0.001 vs CALR Recombinant, ^▲^ *P* < 0.05 vs Control, ^▲▲▲^ *P* < 0.001 vs Control.

Western blot and immunofluorescence analyses revealed that, compared with NC group, the knockdown of TMCO1 led to a decrease in the expression of CALR and Vimentin. In contrast, the CALR recombinant protein, when compared to the control group, significantly increased the expression levels of TMCO1 and Vimentin. Furthermore, the co-treatment with CALR recombinant protein and TMCO1 siRNA resulted in a decrease in the expression of CALR and Vimentin, as compared to the group treated with CALR recombinant protein alone (Figure 7a–l). Additionally, co-immunoprecipitation experiments confirmed an interaction between TMCO1 and CALR (Figure S1).

## 4 Discussion

In the United States alone, over 160,000 new cases of prostate cancer (PCa) are diagnosed annually, with a significant proportion presenting some degree of metastasis [11]. Despite therapeutic interventions, the prognosis for metastatic PCa remains grim, often correlating with a substantial decrease in overall survival [12]. Through analysis of the relationship between TMCO1 and CALR, as well as the pathological characteristics of PCa, and the molecular mechanism affecting its metastasis, we demonstrated that TMCO1 and CALR played vital role in the metastasis of PCa cells, and knockout TMCO1 expression can reverse the effect of CALR recombinant protein. It was discovered that there is a connection between TMCO1 and CALR that influences PCa metastasis via regulating calcium homeostasis.

TMCO1, a transmembrane protein resident in the ER, is recognized as an ER calcium overload-activated calcium channel [13]. It is notably and highly expressed in the thymus, prostate, and testis [14]. TMCO1 plays a critical role in maintaining ER calcium homeostasis by reversibly binding to and releasing calcium ions, thereby detecting and managing excessive calcium levels [5]. Upon encountering an elevation in ER Ca^2+^ concentration, TMCO1 is activated, transitioning from dimers to tetramers, which facilitates the release of the surplus Ca^2+^, averting an overaccumulation of intracellular calcium [15]. It has been found that TMCO1 deficiency can compromise mitochondrial function [16]. The deletion or down-regulation of the TMCO1 gene can trigger a cascade of cellular responses that lead to calcium overload, cytosolic calcium imbalance, and aberrant calcium signaling within the ER. Such dysregulation of intracellular calcium homeostasis can precipitate ER stress and disrupt calcium signal transduction pathways, which are recognized as contributing factors to the process of tumorigenesis [17]. Notably, research has indicated a correlation between the downregulation of TMCO1 and an adverse prognosis in patients with bladder urothelial carcinoma (UBUC) [18]. In glioma research, the upregulation of TMCO1 has been shown to facilitate epithelial-mesenchymal transition (EMT) in U87 and U251 cell lines, thereby enhancing cell migration and invasion [19]. Furthermore, the interaction between iASPP and TMCO1 could offer novel therapeutic strategies for cancer treatment. By specifically suppressing iASPP expression, the levels of TMCO1 protein may be diminished, calcium ion homeostasis could be reestablished, and consequently, tumor progression might be curtailed [20]. Conversely, the knockdown of TMCO1 exerts an inhibitory effect on these processes The relationship between TMCO1 and PCa has not been reported. Research indicates that calcium ion regulation has a “double-sided role” in cancer, with both promoting and inhibiting it. This study indicated that the overexpression of TMCO1 in prostate cancer tissues was closely related to the invasion and metastasis of prostate cancer. These findings underscore the potential of TMCO1 as a diagnostic biomarker and its relevance in the clinical progression of prostate cancer. Interestingly, TMCO1 gene knockout reduced the invasion and migration ability of prostate cancer cells, concurrent with reduced ER stress, mitochondrial membrane potential, and calcium ion levels. The role of TMCO1 in tumor cells is complex and varied, and it may play different roles in different tumor types. In our study, TMCO1 plays an oncogenic role in tumorigenesis, potentially through the modulation of calcium ion homeostasis, which in turn affects the biological behaviors of cancer cells.

CALR, an ER protein with a crucial role in calcium ion binding and protein folding, has been implicated in various cellular processes [9]. Its presence in the nucleus further suggests that it may be involved in transcriptional regulation. CALR binds to the synthetic peptide KLGFKR, which closely resembles an amino acid sequence in the DNA-binding domain of the nuclear receptor superfamily [9]. An bioinformatics analysis of CALR data from public databases reveals that mutations of CALR were widely present in tumors, indicating a broad association with various cancers [21]. Prior research has established an interaction between CALR and the NF-κB signaling pathway. Specifically, elevated CALR expression has been demonstrated to enhance lung cancer cell proliferation by activating the NF-κB signaling [7]. CALR is overexpressed in breast cancer, and its knockdown can affect the spread of the tumor [22]. Evidence suggests that CALR expression is elevated in gastric cancer compared to normal tissue levels, and this increase is associated with lymph node metastasis as well as overall metastatic potential. And the elevated expression of CALR has been demonstrated to enhance the migratory capacity of gastric cancer cells in both *in vivo* and *in vitro* studies [23]. Numerous investigations have demonstrated the influence of CALR on the proliferative and metastatic capabilities of PCa. Alur et al. [24] studies showed that CALR is highly expressed in prostate epithelial cells and is regulated by androgens and a downregulation of CALR expression has been observed in certain PCa specimens. Furthermore, Alur proposes that CALR may play an inhibitory role in the growth and metastasis of PCa. Recent studies have shown that CALR modulates the expression of β1-integrin, impacting PCa metastasis through diverse regulatory mechanisms [25]. Additionally, Zhu has suggested that CALR mediates androgen-induced regulation of calcium sensitivity in LNCaP cells [26]. In the study, it was discovered that CALR can regulate the invasion and migration of prostate cancer cells, and can regulate mitochondrial membrane potential, calcium ion level, and ER stress, and the expression of Vimentin. Our study delineates a pivotal role for CALR in the advancement of tumorigenesis. It is speculated that CALR may affect the metastasis of prostate cancer through calcium ion level and ER stress. The experimental outcomes demonstrated that the knockdown of TMCO1 could reverse the effect of CALR in inducing prostate cancer cell metastasis. This study further demonstrated the regulatory mechanism of TMCO1 and CALR in the metastasis of prostate cancer. Furthermore, our analysis of the TCGA database revealed that both TMCO1 and CALR are upregulated in PCa, with a significant correlation observed between their expression levels. Co-immunoprecipitation results demonstrated a significant interaction between the TMCO1 and CALR. The above results indicate that TMCO1 may regulate the invasion and metastasis of prostate cancer cells, as well as mitochondrial membrane potential, calcium level, and Vimentin expression through CALR.

The analysis of clinical specimens showed that TMCO1 and CALR were overexpressed in PCa and correlated with Gleason score, lymphatic metastasis, invasion depth, and clinical stage and survival. After that, we looked at how recombinant protein CALR and TMCO1 knockdown affected human PCa cells and found that the migration, invasion and proliferation abilities of DU145 and PC-3 were significantly enhanced after CALR recombinant protein, and also had an impact on the intensity of intracellular calcium ions, ER probe and mitochondrial fluorescence probe. Knockdown of TMCO1 significantly inhibited these abilities in DU145 and PC-3 cells and reversed the induction effect of CALR recombinant protein. Most importantly, the knockout of TMCO1 dramatically decreased CALR expression.

In summary, our study delineates the multifaceted roles of TMCO1 and CALR in prostate cancer, underscoring the potential of modulating calcium signaling pathways as a novel therapeutic approach. Although we have shown that interfering with TMCO1 can reverse the induction of CALR recombinant protein through cell experiments, more research is required to determine the precise mechanism by which TMCO1 and CALR effect PCa metastasis. Given their roles in calcium ion homeostasis, the influence of TMCO1 and CALR on the tumor immune microenvironment deserves deeper examination. Later on, a PCa cell subcutaneous tumor model in nude mice can be established.

## 5 Conclusions

In conclusion, TMCO1 plays a key role in the metastasis mechanism of prostate cancer. TMCO1 and CALR can regulate mitochondrial membrane potential, ER stress, and calcium ion level can affect PCa metastasis. The inhibition of TMCO1 expression can reverse the effect of CALR induction, providing a theoretical reference for revealing the metastasis mechanism of prostate cancer cells.

## Supplementary Material

Supplementary Figure
