# Supplementary material for: The relationship between TMCO1 and CALR in the pathological characteristics of prostate cancer and its effect on the metastasis of prostate cancer cells
Source: Open Life Sci. 2024 Oct 29;19(1):20220972. doi: 10.1515/biol-2022-0972 (PMC11524394; doi:10.1515/biol-2022-0972)
Supplement: Supplementary Figure [file biol-2022-0972-sm.pdf]

Supplementary material

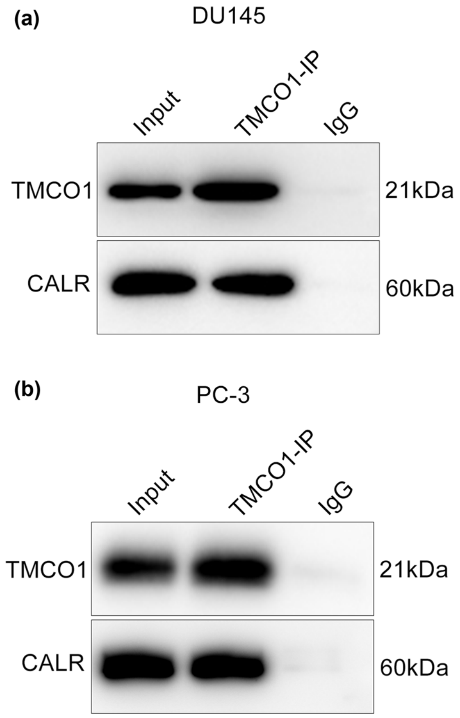

Figure S1: The relationship between TMCO1and CALR in prostate cancer cells. (a) and (b) Co-IP assay verified the binding between TMCO1 and CALR in DU145 and PC-3 cells.
